# Deep single-cell RNA sequencing data of individual T cells from treatment-naïve colorectal cancer patients

**DOI:** 10.1038/s41597-019-0131-5

**Published:** 2019-07-24

**Authors:** Yuanyuan Zhang, Liangtao Zheng, Lei Zhang, Xueda Hu, Xianwen Ren, Zemin Zhang

**Affiliations:** 1 School of Life Sciences and BIOPIC, Peking University, Beijing, 100871 China; 2 Beijing Advanced Innovation Centre for Genomics, Peking-Tsinghua Centre for Life Sciences, Peking University, Beijing, 100871 China

**Keywords:** RNA sequencing, Colorectal cancer, Immunization, Cancer immunotherapy

## Abstract

T cells, as a crucial compartment of the tumour microenvironment, play vital roles in cancer immunotherapy. However, the basic properties of tumour-infiltrating T cells (TILs) such as the functional state, migratory capability and clonal expansion remain elusive. Here, using Smart-seq2 protocol, we have generated a RNA sequencing dataset of 11,138 T cells isolated from peripheral blood, adjacent normal and tumour tissues of 12 colorectal cancer (CRC) patients, including 4 with microsatellite instability (MSI). The dataset contained an expression profile of 10,805 T cells, as well as the full-length T cell receptor (TCR) sequences of 9,878 cells after quality control. To facilitate data mining of our T cell dataset, we developed a web-based application to deliver systematic interrogations and customizable functionalities (http://crctcell.cancer-pku.cn/). Functioning with our dataset, the web tool enables the characterization of TILs based on both transcriptome and assembled TCR sequences at the single cell level, which will help unleash the potential value of our CRC T cell data resource.

## Abstract (summary)

**Table:** 

Design Type(s)	transcription profiling design • disease analysis objective
Measurement Type(s)	transcription profiling assay
Technology Type(s)	RNA sequencing
Factor Type(s)	Microsatellite Instability • age • sex • experimental condition • tumor stage
Sample Characteristic(s)	Homo sapiens • lymphocyte

Machine-accessible metadata file describing the reported data (ISA-Tab format)

## Background & Summary

CRC is among the common causes of cancer-related mortality worldwide^1,2^. While immune checkpoint blocking antibodies (ICBs) have shown impressive clinical benefits in cancers^3–6^, their benefits are highly uneven among CRC patients. Remarkably, only CRC patients with MSI showed pronounced responses to ICBs, while patients with microsatellite stability (MSS) derived no benefit^7,8^. The underlying mechanisms of such discrimination remain elusive. T cells play vital roles in killing malignant cells and are associated with responses to ICB-treatment^9,10^. It is thus important to understand the cellular underpinnings of TILs in CRC.

**Fig. 1:**
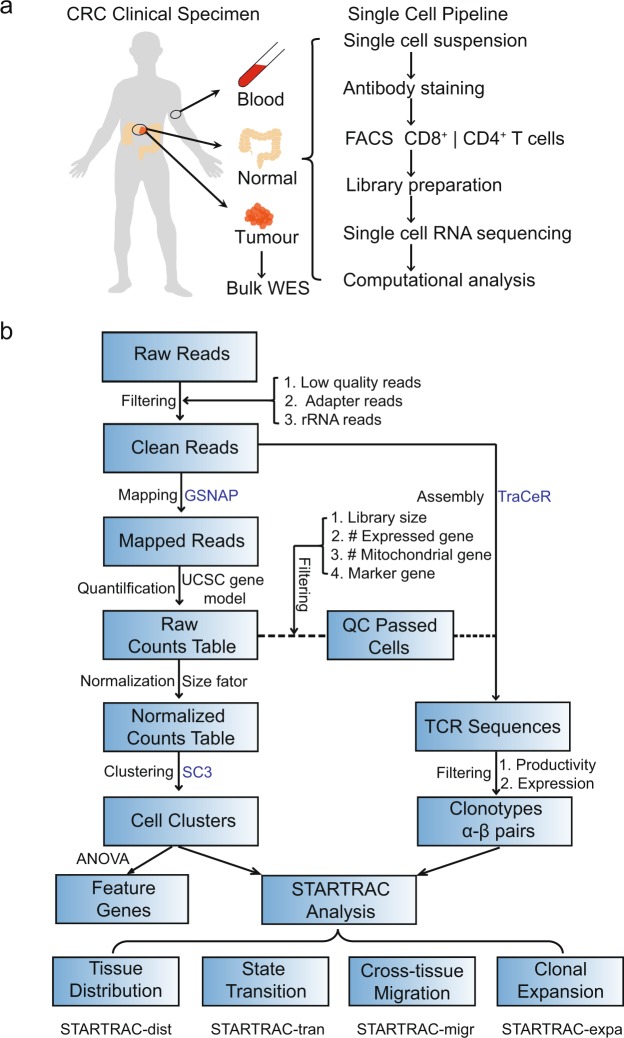
Schematic overview of the study design and analysis pipeline. (**a**) The experimental flowchart of this study. (**b**) The bioinformatics pipeline used for data analysis. Softwares used in each steps were labelled in blue. WES, whole exome sequencing; DEG, differentially expressed gene; dist, tissue distribution; expa, clonal expansion; migr, cross-tissue migration; tran, developmental transition.

Single cell transcriptome analysis has become a compelling approach to decipher the properties of TILs, due to its ability to quantify gene expression and assemble TCR sequences simultaneously. In our recent *Nature* paper, we have performed single cell RNA sequencing of 11,138 T cells isolated from peripheral blood, adjacent normal and tumour tissues of 12 treatment-naïve CRC patients (Fig. 1a and Table 1), and developed STARTRAC (single T cell analysis by RNA sequencing and TCR tracking) indices to analyse the dynamic relationships among 20 identified T cell subsets^11^. Here, we provide the detailed description of our dataset and present a webserver to deliver comprehensive and customizable analyses.

**Table 1:** Clinical characteristics of 12 CRC patients.

Patient ID	Age	Gender	Histological type^a^	Stage	Tumour size	MSI status^b^	TNM Classification	Grade
P0701	68	Female	Rectum ADC	I	1 × 0.8 cm	MSS	1,0,0	Well- differentiated
P1012	35	Female	Colon ADC	IIIC	7 × 6 cm	MSS	4,2,0	Low-differentiated
P1207	66	Female	Colon ADC	II	6 × 6 cm	MSS	4,0,0	Moderate-differentiated
P1212	42	Female	Colon ADC	II	6 × 4 cm	MSS	4,0,0	Low- or moderate- differentiated
P1228	77	Female	Colon ADC	II	4.5 × 4 cm	MSS	4,0,0	Low- or moderate- differentiated
P0215	75	Male	Colon ADC	IV	6.5 × 4 cm	MSS	4,2,1	Low-differentiated
P0309	55	Male	Rectum ADC	IIIC	5 × 4.5 cm	MSS	3,2,0	Moderate- differentiated
P0411	75	Male	Rectum ADC	IIB	6.5 × 3.5 cm	MSS	4,0,0	Moderate- differentiated
P0123	65	Female	Colon ADC	IIIB	11.5 × 7 cm	MSI	4,1,0	Moderate- differentiated
P0413	82	Female	Colon ADC	IIIB	10 × 10 cm	MSI	4,1,0	Moderate- differentiated
P0825	83	Female	Colon ADC	IIB	9 × 4 cm	MSI	4,0,0	Low- differentiated
P0909	45	Male	Colon ADC	IIIB	6 × 4 cm	MSI	3,1,0	Low- differentiated

^a^ADC, adenocarcinoma.

^b^MSS, microsatellite stability; MSI, microsatellite instability.

**Fig. 2:**
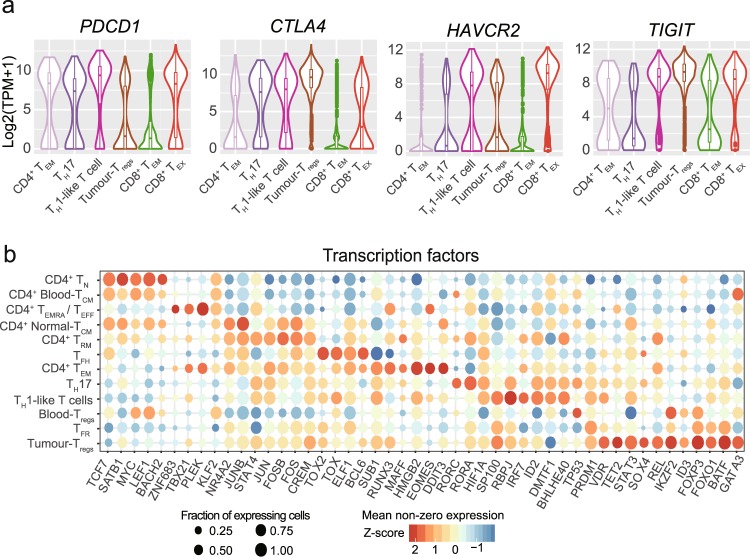
Expression patterns of selected genes. (**a**) Violin plots showing the expression distributions of known immunotherapy targets in tumour-enriched T cell clusters. (**b**) Bubble plots depicting expressions of transcription factors in different CD4^+^ T cell clusters.

The dataset contained an average of 1.25 million uniquely mapped read pairs per cell, with an average mapping rate of 96.6% (Online-only Table 1). After quality control, we obtained an expression profile of 12,547 genes for 10,805 cells, with an average of 3,182 genes detected per cell (Online-only Table 1). The expression data could be used to elucidate the expression distributions of genes including those currently pursued as immunotherapy targets in clinical trials (Fig. 2a), illuminating the potentially modulated T cell populations with different immunotherapies. Furthermore, the dataset can serve as a resource for further T cells exploration including the identification of novel regulatory mechanisms by depicting the specific expression patterns of transcription factors (Fig. 2b).

**Fig. 3:**
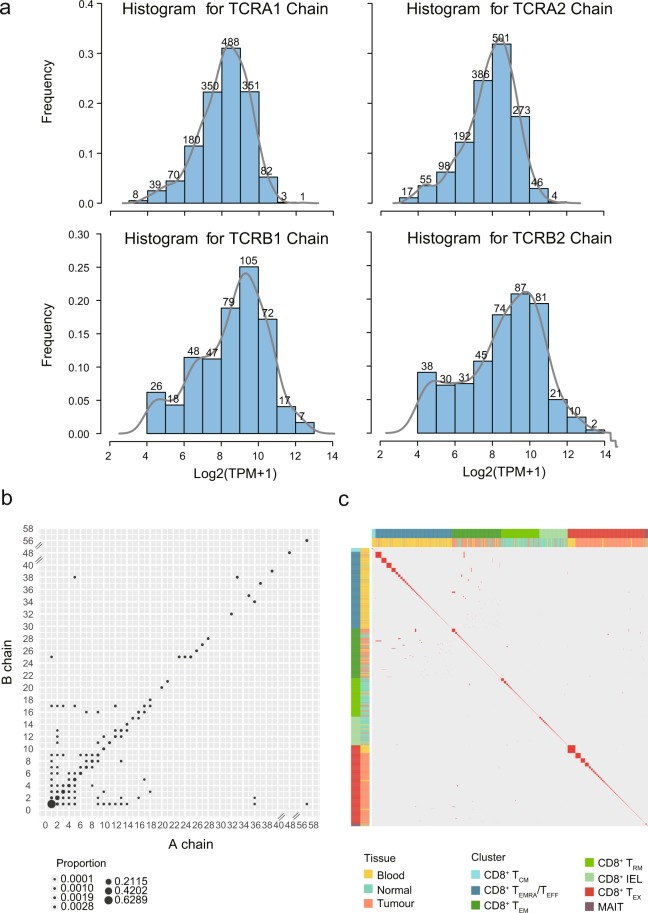
The TCR profile of single T cells. (**a**) The abundance distributions of TCR α- or β-chain. The gray lines represent the fitting values. (**b**) The relationship between the degrees of recurrent usage of various TCR α-chains with that of β-chains. Each dot represents a group of TCR α/β allele expressed in a given number of cells. Dot size represents the proportion of such group in all TCR chains detected. (**c**) TCR sharing patterns of different CD8^+^ T cell clusters enriched in different tissues.

TCR sequences, composed of α- and β-chains, play major roles in the selection and activation of T cells^12^. Both α- and β-chains contribute to the determination of TCR antigen specificity, and different T cells with the same TCR could be functionally distinct^13^. To uncover information about T cell ancestry and clonality, we obtained full-length TCR sequences of 91.4% (9,878/10,805) cells with at least one pair of productive α-β chains after eliminating non-productive alleles or low-abundance TCRs (Fig. 3a and Supplementary File 1). Accordingly, T cells with identical TCRs were defined to be from the same clonotype, and a total of 7,274 clonotypes were obtained (Supplementary File 1). Indeed, a strong correlation was observed between the recurring frequencies of α-chains and that of β-chains, indicating a common ancestral cell of origin (Fig. 3b).

The TCR sequences can be utilized to delineate TCR sharing patterns of both inter/intra-tissues and inter/intra-clusters (Fig. 3c), shedding light on the properties of T cells including clonal expansion, developmental transition and cross-tissue migration. Furthermore, TCR sequences, as well as the transcriptome data elucidating T cell functions, could serve as a data resource for the discovery of antigen specificity in therapeutic applications^14^.

In our related work, we have revealed important insights of the T cell biology based on STARTRAC indices^11^. For instance, tumour-resident CD8^+^ effector memory and dysfunctional T cells showed mutually exclusive developmental transition patterns, suggesting a TCR-based cell fate decision. In addition, we found that a special subset of *IFNG*^+^ T_H_1-like T cells with *CXCL13*^+^*BHLHE40*^+^ were preferentially enriched in MSI tumours, which might contribute to the favourable responses of MSI patients to ICBs.

**Fig. 4:**
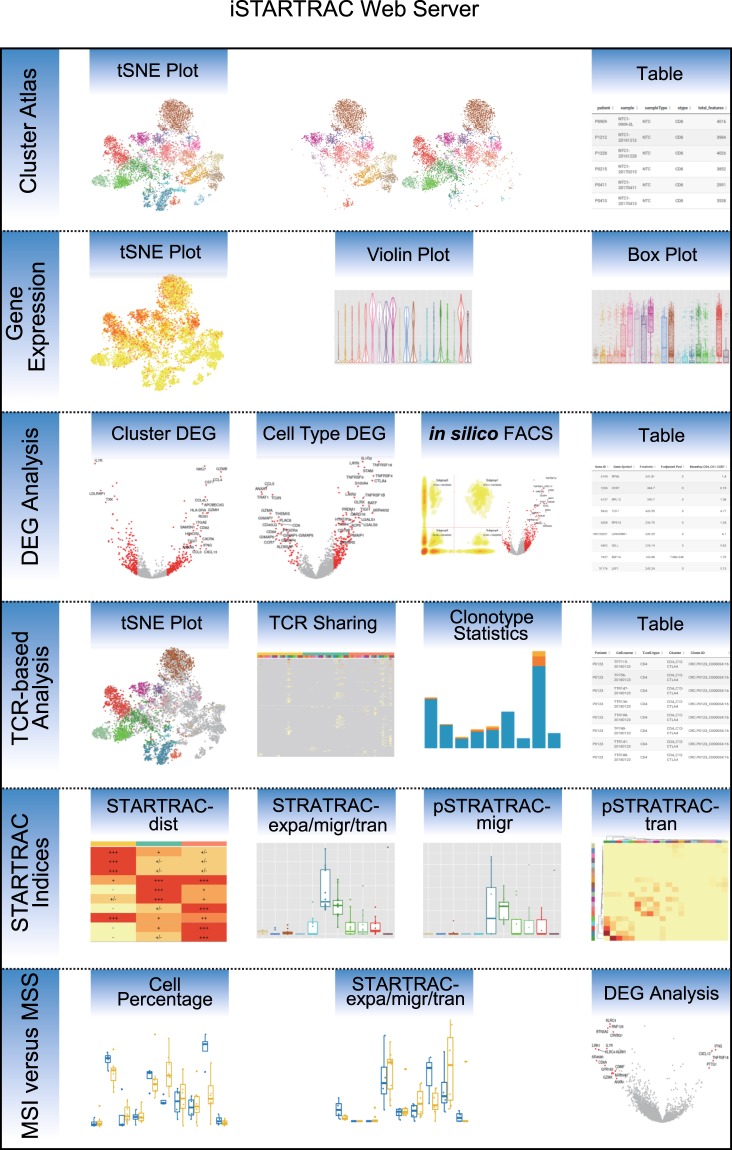
Schema describing the key functionalities of the iSTARTRAC web server. iSTARTRAC provides six functional modules including cluster atlas, gene expression, DEG analysis, TCR-based analysis, STRATRAC indices and MSI versus MSS. Each module implements several customizable analyses for user input samples.

While some discoveries have been made, the unprecedented data resource of CRC T cells is still attractive to many biologists. To facilitate data mining of our T cell dataset, we developed iSTARTRAC (the interactive platform of STARTRAC), a web server to deliver customizable functionalities for further T cell investigation. iSTARTRAC provides key functions including cluster visualization, gene expression demonstration, differential expression analysis, TCR sharing illustration and discrimination of differences between MSI and MSS patients (Fig. 4).

The comprehensive and customizable analyses with simple clicking through iSTARTRAC could greatly facilitate data reuse in the field of cancer immunology, and the accompanying scientific discussion will further expedite the process of therapeutic discovery and understanding the mechanism of immunotherapies with respect to T cell functions.

## Methods

These methods are expanded version of descriptions in our related work^11^, which provided detailed descriptions of experimental procedures including human specimens, single cell collection, cell sorting, reverse transcription, amplification and sequencing, and those of computational processing including quality control, data processing, TCR assembly, unsupervised clustering and definition of STARTRAC indices^11^. While most part of the methods described here was cited from that report, we specifically aim to emphasize the samples and the methods used to generate the single cell RNA-seq data.

### Clinical human specimens

Twelve patients with CRC were enrolled and pathologically diagnosed with colorectal adenocarcinoma at Peking University People’s Hospital. All patients in this study provided written informed consent for sample collection and data analyses. This study was approved by the Research and Ethical Committee of Peking University People’s Hospital and complied with all relevant ethical regulations.

The patients included eight with MSS (P0701, P1012, P1207, P1212, P1228, P0215, P0411 and P0309) and four with MSI (P0123, P0909, P0825 and P0413) status. Among these 4 MSI patients, 3 had positive lymph nodes (P0123, P0413 and P0909), two of them had poorly-differentiated disease (P0825 and P0909), and none of them had distal metastasis. There were eight females and four males, and the median age of diagnosis was 67, ranging from 35 to 82. Among these 12 patients, one was diagnosed at stage I, five at stage II, five at stage III, and one at stage IV, which was classified according to the guidance of AJCC version 8. None of them were treated with chemotherapy or radiation prior to tumour resection. The available clinical characteristics are summarized in Table 1.

### Sample collection and preparation

Fresh tumour and adjacent normal tissue samples (at least 2 cm from matched tumour tissues) were surgically resected from the above-described patients. Patients P0701, P0909, P1212, P1228, P0215, P0411, P0413, P0825, P0123 and P0309 had peripheral blood and paired tumour and adjacent normal tissues, whereas patients P1012 and P1207 had only fresh tumour tissue and matched peripheral blood.

Tumours and adjacent normal tissues were cut into approximately 1-mm^3^ pieces in the RPMI-1640 medium (Invitrogen) with 10% fetal bovine serum (FBS; Sciencell), and enzymatically digested with MACS Tumour Dissociation Kit (Miltenyi Biotec) for 30 min on a rotor at 37 °C, according to the manufacturer’s instruction. The dissociated cells were subsequently passed through a 40-µm cell-strainer (BD) and centrifuged at 400 g for 10 min. After the supernatant was removed, the pelleted cells were suspended in red blood cell lysis buffer (Solarbio) and incubated on ice for 2 min to lyse red blood cells. After washing twice with PBS (Invitrogen), the cell pellets were re-suspended in sorting buffer (PBS supplemented with 1% FBS). PBMCs were isolated using HISTOPAQUE-1077 (Sigma-Aldrich) solution as previously described^15^. In brief, 3 ml of fresh peripheral blood was collected before surgery in EDTA anticoagulant tubes and subsequently layered onto HISTOPAQUE-1077. After centrifugation, lymphocyte cells remained at the plasma–HISTOPAQUE-1077 interface and were carefully transferred to a new tube and washed twice with PBS. Red blood cells were removed via the same procedure described above. These lymphocytes were re-suspended in sorting buffer.

### Single-cell sorting, reverse transcription, amplification and sequencing

Single-cell suspensions were stained with antibodies against CD3, CD4, CD8 and CD25 (anti-human CD3, UCHT1; anti-human CD4, OKT4; anti-human CD8, OKT8; anti-human CD25, BC96; eBioscience) for fluorescence-activated cell sorting (FACS), performed on a BD Aria III instrument. Single cells of different subtypes including cytotoxic T (T_C_) cells, T helper (T_H_) cells and regulatory T (T_reg_) cells were enriched by gating 7AAD^−^CD3^+^CD8^+^, 7AAD^−^CD3^+^CD4^+^CD25^−/+^ and 7AAD^-^CD3^+^CD4^+^ CD25^++^ T cells, respectively, and sorted into 96-well plates (Axygen) chilled to 4 °C, prepared with lysis buffer with 1 µl 10 mM dNTP mix (Invitrogen), 1 µl 10 µM Oligo dT primer, 1.9 µl 1% Triton X-100 (Sigma), and 0.1 µl 40 U µl-1 RNase Inhibitor (Takara). The single-cell lysates were sealed and stored frozen at −80 °C immediately. Single-cell transcriptome amplifications were performed according to the Smart-Seq2 protocol^15,16^. The External RNA Controls Consortium (ERCC; Ambion; 1:4,000,000) was added into each well as the exogenous spike-in control before the reverse transcription. The amplified cDNA products were purified with 1× Agencourt XP DNA beads (Beckman). A procedure of quality control was performed following the first round of purification, which included the detection of CD3D by qPCR (forward primer, 5′-TCATTGCCACTCTGCTCC-3′; reverse primer, 5 primer, 5′-TCATTGCCACT) and fragment analysis by analyser AATI. For those single-cell samples with high quality after quality control (cycle threshold <30), the DNA products were further purified with 0.5× Agencourt XP DNA beads, and the concentration of each sample was quantified by Qubit HsDNA kits (Invitrogen). Multiplex (384-plex) libraries were constructed and amplified using the TruePrep DNA Library Prep Kit V2 for Illumina (Vazyme Biotech). The libraries were then purified with Agencourt XP DNA beads and pooled for quality assessment by fragment analyser. For all the 12 patients, purified libraries were analysed by an Illumina Hiseq 4000 sequencer with 150-bp pair-end reads. For patient P1207, only CD8^+^ T cells were collected due to the temporary lack of CD4 antibody.

### Bulk DNA isolation and sequencing

Genomic DNA of peripheral blood and tissue samples of patients with CRC were extracted using the QIAamp DNA Mini Kit (QIAGEN) according to the manufacturer’s specification. The concentrations of DNA were quantified using the Qubit HsDNA Kits (Invitrogen) and the qualities of DNA were evaluated with agarose gel electrophoresis. Exon libraries were constructed using the SureSelectXT Human All Exon V5 capture library (Agilent). Samples were sequenced on the Illumina Hiseq 4000 sequencer with 150-bp paired-end reads.

### Multi-colour immunohistochemistry

Opal^TM^ multi-colour immunohistochemistry (IHC) staining were performed with antibodies of rabbit anti-human CD3 (Abcam, clone SP7, 1:400), mouse anti-human CD8 (Abcam, clone 144B, 1:500), rabbit anti-human CD4 (Abcam, clone EPR6855, 1:400) and mouse anti-human FOXP3 (Abcam, clone mAbcam22510, 1:500) to validate the existence of infiltrating T_C_, T_H_ and T_reg_ cells in tumour tissues. The specimens were collected and prepared for the formalin-fixed paraffin-embedded tissues sections as previously mentioned^15^. Antigen was retrieved by AR9 buffer (pH 6.0, PerkinElmer) and boiled in the oven for 15 min. After a pre-incubation with blocking buffer at room temperature for 10 min, the sections were incubated at room temperature for 1 h with aforementioned antibodies. A secondary horseradish peroxidase-conjugated antibody (PerkinElmer) were added and incubated at room temperature for 10 min. Signal amplification was performed using TSA working solution diluted at 1:100 in 1× amplification diluent (PerkinElmer) and incubated at room temperature for 10 min. The multispectral imaging was collected by Mantra Quantitative Pathology Workstation (PerkinElmer, CLS140089) at 20× magnification and analysed by InForm Advanced Image Analysis Software (PerkinElmer) version 2.3. For each patient, a total of 8–15 high-power fields were taken based on their tumour sizes.

### Microsatellite instability testing

DNA purified from tumour tissues using QIAamp DNA Mini Kit (QIAGEN) was subjected to multiplex fluorescent PCRbased assay (Promega) by amplifying seven loci including five mononucleotide repeats (NR21, BAT26, BAT25, NR24 and Mono27) and two pentanucleotide repeats (PentaC and PentaD) and was compared with DNA extracted from matched adjacent normal tissues. Multiplex PCR products were analysed by ABI PRISM 3100 Genetic Analyzer (Applied Biosystems).

### Quality control and preprocessing of single cell RNA-seq data

Low-quality read pairs of single-cell RNA sequencing (scRNA-seq) data were filtered out if at least one end of the read pair met one of the following criteria: (1) ‘N’ bases account for ≥10% of the read length; (2) bases with quality <5 account for ≥50% of the read length; and (3) the read contains adaptor sequence. The filtered read pairs were processed using HTSeqGenie pipeline (R package version 4.8) to obtain the gene expression table. Specially, read pairs were then mapped to human ribosomal RNA (rRNA) sequences (download from RFam database) and the read pairs with both ends unmapped were kept for downstream analysis. Read pairs passing this filter for rRNA were aligned to human reference sequence (hg19) using GSNAP^17^, with parameters ‘–novelsplicing 1 -n 10 -i 1 -M 2’. To calculate the expression levels of genes, the gene model file ‘knownGene.txt’ (30 June 2013 version), downloaded from UCSC, was used. The R function findOverlaps was used to count the number of uniquely mapped read pairs located in each gene and the count table tabulated as genes by cells was used for downstream analysis. The transcripts per million (TPM) table was derived from the count table and the TPM value was calculated by

$$\frac{1{0}^{6}\times {C}_{ij}/length\,of\,gene\,i}{{\sum }_{i}\,{C}_{ij}/length\,of\,gene\,i}$$

where C_ij_ is the count value of gene i in cell j. It should be noticed that the TPM here is a simplified version based on the hypothesis that all mapped reads are approximate the same length.

Low-quality cells were filtered if the library size or the number of expressed genes (counts larger than 0) was smaller than predefined thresholds. Both thresholds were defined as the medians of all cells minus 3× the median absolute deviation. Furthermore, if the proportion of mitochondrial gene counts was larger than 10%, these cells were discarded. Only cells with the average TPM of *CD3D*, *CD3E* and *CD3G* larger than 10 were kept for subsequent analysis. We further identified CD4^+^, CD8^+^, CD4^−^CD8^−^ (double negative) and CD4^+^CD8^+^ (double positive) T cells based on the gene expression data. Given the average TPM of *CD8A* and *CD8B*, one cell was considered as *CD8* positive or negative if the value was larger than 30 or less than 3, respectively; given the TPM of *CD4*, one cell was considered as *CD4* positive or negative if the value was larger than 30 or less than 3, respectively. Hence, the cells can be *in silico* classified as CD4^+^CD8^−^, CD4^−^CD8^+^, CD4^+^CD8^+^, CD4^−^CD8^−^ and other cells that cannot be clearly defined.

While TPM is an intuitive and popular measurement to standardize the total number of transcripts between cells, it is insufficient and could bias downstream analysis because TPM can be dominated by a handful of highly expressed genes. Therefore, we mainly used TPM for preliminary data processing and gene expression visualization. Recently, methods for normalizing scRNA-seq data including scran^18^ have been proposed to implement robust and effective normalization, and thus we used the size-factor normalized read count for main analyses in our study including dimensionality reduction, clustering and finding markers for each cluster.

After discarding genes with average counts of fewer than or equal to 1, the count table of the cells passing the above filtering was normalized by a pooling strategy. We applied the R package scran^18^ in Bioconductor to perform the normalization process. Specifically, cells were pre-clustered using the ‘quickCluster’ function with the parameter ‘method = hclust’. Size factors were calculated using ‘computeSumFactors’ function with the parameter ‘sizes = seq (20,100,by = 20)’ which indicates the number of cells per pool. Raw counts of each cell were divided by their size factors, and the resulting normalized counts were then scaled to log2 space and used for batch correction.

Scran utilizes a pooling strategy implemented in ‘computeSumFactors’ function, in which size factors for individual cells were deconvoluted from size factors of pools. To avoid violating the assumption that most genes were not differentially expressed, hierarchical clustering based on Spearman’s rank correlation was performed with ‘quickCluster’ function first, then normalization was performed in each resulting cluster separately. The size factor of each cluster was further re-scaled to enable comparison between clusters.

To remove the possible effects of different donors on expression, the normalized table was further centred by patient. Thus, in the centred expression table, the mean values of the cells for each patient were zero. A total of 12,548 genes and 10,805 cells were retained in the final expression table. If not explicitly stated, ‘normalized read count’ or ‘normalized expression’ in this study refers to the normalized and centred count data for simplicity.

### Unsupervised clustering analysis of CRC single T cell RNA-seq dataset

The cell clusters used here were the same as defined in our related Nature paper^11^. The expression tables of CD8^+^CD4^−^ T cells and CD8^−^CD4^+^ T cells as defined by the aforementioned *in silico* classification but excluding MAIT cells and iNKT cells, were fed into an iteratively unsupervised clustering pipeline separately. Specifically, given expression table, the top n genes with the largest variance were selected, and then the expression data of the n genes were analysed by single-cell consensus clustering (SC3)^19^. n was tested from 500, 1000, 1500, 2000, 2500 and 3000. In SC3, the distance matrices were calculated based on Spearman correlation and then transformed by calculating the eigenvectors of the graph Laplacian. Then the k-means algorithm was applied to the first d eigenvectors multiple times where d was chosen from 4% to 7% of the total number of input cells. Finally, hierarchical clustering with complete agglomeration was performed on the SC3 consensus matrix and k clusters were inferred. The SC3 parameters k, which was used in the k-means and hierarchical clustering, was tried from 2 to 10. For each SC3 run, the silhouette values were calculated, the consensus matrix was plotted, and cluster specific genes were identified. Such information was used to determine the optimal k and n. Once the stable clusters were determined, the above procedure was iteratively applied to each of these clusters to reveal the sub-clusters. After obtained the stable clusters by SC3, we further redefined the cluster labels of indeterminate cells with the silouatte values less than zero by R package XGBoost^20^. The training datasets were composed of cells with the silouatte >0, while cells to be reclassified with the silouatte <0 were then redefined to clusters with the largest predicting score. The *in silico* classified CD8^+^CD4^−^ MAIT cells had distinct gene expression patterns compared with other CD8^+^CD4^−^ T cells, and were defined as cluster “CD8_C08-SLC4A10”.

When the clustering results were obtained, one-way ANOVA implemented by R function aov was performed to identify the differentially expressed genes among the clusters. R function TukeyHSD was used to identify which cluster pairs showed a significant difference. A gene was defined as being significantly differentially expressed based on the following criteria: 1) adjusted P-value (Benjamini-Hochberg method) of F test less than 0.05; 2) the absolute difference of any one significant cluster pair (P-value of Tukey’s ‘Honest Significant Difference’ method less than 0.01) larger than 1. The significantly differentially expressed genes were categorized in the cluster that showed the highest expression.

The *t*-SNE method implemented in R package Rtsne was used for clustering visualization. To visualize the cell density on the *t*-SNE plot, kernel density estimation was performed using R function kde (ks package), and the contour lines encompassing the top 10%, 20%, …90% cells with highest densities were shown. A total of 8,530 T cells, including 3,628 CD8^+^CD4^−^ and 4,902 CD8^−^CD4^+^ T cells with clustering definitions, were used in the *t*-SNE projection. Other cells such as CD8^+^CD4^+^ and CD8^−^CD4^−^ T cells were not included in this visualization.

### Analysis pipelines of bulk exome sequencing data

The bulk exome sequencing data were cleaned following the same procedure for the scRNA-seq data processing. The cleaned read pairs were then processed according to the BWA-Picard/ Genome Analysis Toolkit (GATK)-Strelka pipeline. In brief, the cleaned read pairs were aligned to human genome reference version b37 (downloaded from ftp://ftp.broadinstitute.org:/bundle) by the BWA-MEM algorithm^21^. The alignments were then sorted and de-duplicated by Picard (Broad Institute). GATK^22^ was used to realign multiple reads around putative INDEL by Smith–Waterman alignment algorithm and re-calibrate base quality. The analysis-ready bam files were input into the GATK UnifiedGenotyper module to call SNP/INDEL and into Strelka^23^ to call somatic SNV/INDEL and into ADTEx^24^ (version 1.0.4) to call somatic copy number alterations. The mutations were annotated with ANNOVAR^25^.

### TCR assembly

TraCeR^26^ was used to deduce the TCR sequences of each cell. The outputs of TraCeR include the assembled nucleotide sequences for both α and β chains, the coding potential of the nucleotide sequences (that is, productive or not), the translated amino acid sequence, the CDR3 sequences and the estimated TPM value of α or β chains. Only cells with TPM values larger than 10 for the α chain and larger than 15 for the β chain were kept. For cells with two or more α or β chains assembled, the α–β pair that was productive and of the highest expression level was defined as the dominant α–β pair in the corresponding cell. If two cells had identical dominant α–β pairs, the dominant α–β pair was identified as clonal TCRs.

To integrate with the gene expression data, the TCR-based analysis was performed only for cells that passed the aforementioned quality control pipeline (total 10,805). Thus, 9,878 cells with TCR information were used in the integrative analysis^27^ (Supplementary File 1). If one cell had an α chain composed of V segment TRAV1-2 and one of the following J segments (TRAJ33, TRAJ20 and TRAJ12), the cell was classified as a MAIT cell^28^. If the α chain of one cell was rearranged by V segment TRAV10 and J segment TRAJ18, the cell was classified as an invariant natural killer T cell^29^. In the 9,878 cells with at least one pair of productive α and β chains, only 3 cells were identified as invariant natural killer T cells, and 102 cells were identified as MAIT cells, including 71 CD8^+^CD4^−^ T cells classified *in silico*.

### Definition of STARTRAC indices

We present STRATRAC as a framework, defined by four indices, to analyse different aspects of T cells based on paired single cell transcriptomes and TCR sequences. The first index, named as STARTRAC-dist (STARTRAC-distribution), utilizes the ratio of observed over expected cell numbers in tissues to measure the enrichment of T cell clusters across different tissues. Given a contingency table of T cell clusters by tissues, we first apply Chi-squared test to evaluate whether the distribution of T cell clusters across tissues significantly deviates from random expectations. We then calculate the STARTRAC-dist index for each combination of T cell clusters and tissues according the following formula:

$${I}_{dist}^{STARTRAC}={R}_{o/e}=\frac{Observed}{Expected}$$

where *R*_*o*/*e*_ is the ratio of observed cell number over the expected cell number of a given combination of T cell cluster and tissue. The expected cell number for each combination of T cell clusters and tissues are obtained from the Chi-squared test. ${I}_{dist}^{STARTRAC}$ can indicate whether cells of a certain cluster are enriched (R_o/e_ > 1) or depleted (R_o/e_ < 1) in a specific tissue.

The other three STARTRAC indices, STARTRAC-expa (STARTRAC-expansion), STARTRAC-migr (STARTRAC-migration) and STARTRAC-tran (STARTRAC-transition), are designed to measure the degree of clonal expansion, tissue migration, and state transitions of T cell clusters upon TCR tracking, respectively. The MAIT cells were not included in these types of analyses because they have distinct TCRs. For STARTRAC-expa, which uses the standard TCR clonality measurement^30^ but is specifically applied to different T cell clusters in our analyses, we first adopt the normalized Shannon entropy to calculate the evenness of the TCR repertoire of the given T cell cluster and then define the STARTRAC-expa index as 1-evenness. Mathematically, the STARTRAC-expa index of a specific cluster with *N* clonotypes is defined by the following formula:

$${I}_{expa}^{STARTRAC}=1-eveness=1-\frac{-{\sum }_{i=1}^{N}{p}_{i}{{\rm{log}}}_{2}{p}_{i}}{{{\rm{log}}}_{2}N}$$

where *p*_*i*_ is the cell frequency of clonotype *i* in the cluster, and a clonotype is defined by identical, full-length, paired α and β TCR chains. STARTRAC-expa ranges from 0 to 1, with 0 indicating no clonal expansion for each clonotype while 1 indicating that the cluster is composed of only one clonally expanded clonotype, with high STARTRAC-expa indicating high clonality.

For T cells with identical TCR clonotypes, even if they are present in different tissues or in different development states, logically they could be likely derived from a single naïve T cell, clonally expanded initially at one location and migrated across tissues or have undergone state transitions. Based on this principle, we define STARTRAC-migr and STARTRAC-tran to evaluate the extent of tissue migration and state transition of each clonotype, respectively. For each clonotype, given its distribution across tissues (peripheral blood, adjacent normal mucosa and tumour), we define its STARTRAC-migr index ${I}_{migr}^{t}$ as:

$${I}_{migr}^{t}=-\sum _{j=1}^{J}{p}_{j}^{t}{{\rm{log}}}_{2}{p}_{j}^{t}$$

where ${p}_{j}^{t}$ is the ratio of the number of cells with TCR clonotype *t* in tissue *j* to the total number of cells with TCR clonotype *t* and ${\sum }_{j=1}^{J}{p}_{j}^{t}=1$. For two T cell clusters with similar clonal expansion and clonal size, the one with clonal cells broadly distributed in various tissues would likely be more mobile. Similarly, its STARTRAC-tran index ${I}_{tran}^{t}$ can be defined as:

$${I}_{tran}^{t}=-\sum _{k=1}^{K}{p}_{k}^{t}{{\rm{log}}}_{2}{p}_{k}^{t}$$

where ${p}_{k}^{t}$ is the ratio of the number of cells with TCR clonotype *t* in cluster *k* to the total number of cells with TCR clonotype *t*, ${\sum }_{k=1}^{K}{p}_{k}^{t}=1$, and *K* is the total number of cell clusters. The input of STARTRAC-migr is the observed cell frequency across tissues of a certain clonotype, while the input of STARTRAC-tran is the observed cell frequency across cell clusters of a certain clonotype. By contrast, the input of STARTRAC-expa is the observed cell frequency across clonotypes of a certain cell cluster, and the input for the traditional TCR clonality measure is the observed sequence frequency across a TCR repertoire of a given sample.

After the extent of tissue migration of each clonotype is quantified by STARTRAC-migr, given a cluster with total *T* clonotypes, the STARTRAC-migr index at the cluster level ${I}_{migr}^{STARTRAC}$ can be defined as the weighted average of all TCR clonotype migration indices contained in the cluster:

$${I}_{migr}^{STARTRAC}=\sum _{t=1}^{T}{p}_{cls}^{t}{I}_{migr}^{t}$$

where ${p}_{cls}^{t}$ is the ratio of the number of cells with clonotype *t* in cluster *cls* to the total number of cells in cluster *cls*.

Similarly, when the extent of state transition of each clonotype is quantified by STARTRAC-tran, given a cluster with total *T* clonotypes, the STARTRAC-tran index at the cluster level can be defined as the weighted average of all TCR clonotypes state transition indices contained in the cluster:

$${I}_{tran}^{STARTRAC}=\sum _{t=1}^{T}{p}_{cls}^{t}{I}_{tran}^{t}$$

where ${p}_{cls}^{t}$ is the ratio of the number of cells with clonotype *t* in cluster *cls* to the total number of cells in cluster *cls*.

Besides the overall evaluation of the extents of migration and state transitions by STARTRAC-migr and STARTRAC-tran, we also define pairwise STARTRAC-migr (pSTARTRAC-migr) and STARTRAC-tran (pSTARTRAC-tran) indices for precise quantification. For example, given a clonotype *t* and two tissue types (e.g., blood and tumour), the pSTARTRAC-migr index ${p}^{{I}_{migr}^{t}}$ is calculated by the following formula:

$${p}^{{I}_{migr}^{t}}=-\sum _{j=1}^{2}{p}_{j}^{t}{{\rm{log}}}_{2}{p}_{j}^{t}$$

where ${p}_{j}^{t}$ is the ratio of the number of cells with TCR clonotype *t* in tissue *j* to the total number of cells with TCR clonotype *t* in tissues 1 and 2 (i.e., blood and tumour), and ${\sum }_{j=1}^{2}{p}_{j}^{t}=1$. In other words, pSTARTRAC-migr uses the same formula as STARTRAC-migr but limits the number of tissues to two and the frequencies of cells between two specified tissues are re-calculated. Likewise, given a clonotype *t* and two T cell clusters (e.g., T_EM_ and T_EX_), the pSTARTRAC-tran index ${p}^{{I}_{tran}^{t}}$ is calculated by the following formula:

$${p}^{{I}_{tran}^{t}}=-\sum _{k=1}^{2}{p}_{k}^{t}{{\rm{log}}}_{2}{p}_{k}^{t}$$

where ${p}_{k}^{t}$ is the ratio of the number of cells with TCR clonotype *t* in cluster *k* to the total number of cells with TCR clonotype *t* in clusters 1 and 2 (i.e., T_EM_ and T_EX_), and ${\sum }_{k=1}^{2}{p}_{k}^{t}=1$. Thus, pSTARTRAC-tran uses the same formula as STARTRAC-tran but limits the number of clusters to two and the frequencies of cells between the two specified clusters are re-calculated. Once pairwise STARTRAC-migr and STARTRAC-tran for clonotypes are obtained, the corresponding indices for clusters are calculated via weighted average according to their clonotype compositions.

### Summary of scRNA-seq data and bioinformatics workflow used for data processing

For all the 12 patients, a total of 35.5 G raw reads and 5.4 T raw bases were obtained after sequencing. After preprocessing, we obtained 32.5 G high-quality reads with an average high-quality rate of 91.3% (Online-only Table 1). Accordingly, we summarized the data processing procedures and tools used in each step in a flowchart, consisting of quality control filtering, TCRs assembly, expression quantification, data normalization and downstream analyses (Fig. 1b).

## Data Records

As described in our related research paper^11^, the raw sequencing data have been deposited in the European Genome-phenome Archive database under study accession id EGAS00001002791 and dataset accession id EGAD00001003910^31^, which are available in FASTQ file format upon request and approval. The DATA ACCESS AGREEMENT is provided at https://github.com/zhangyybio/single-T-cell-data-access. Applicants can request access to the data by directly downloading it or by sending an email to cancerpku@pku.edu.cn. The process that is used to approve an application includes verifying the institution, participants and research purposes of the application, and the authorization by EGA. In general this process will take about two weeks. In principal, any academic research institutions complying with the laws and bioethic regulation policies of China will be approved. The publication moratorium described in the Data Access Agreement officially expires concurrent with publication of this Data Descriptor. The processed gene expression data were deposited in the Gene Expression Omnibus database under accession id GSE108989^32^. The clinical data recording available clinical characteristics of the collected 12 CRC patients are summarized in Table 1 and the genomic features are summarized in Table 2 and Online-only Table 2. Online-only Table 3 lists the DNA fragment sizes of short tandem repeat loci from tested patients in microsatellite instability testing experiment. Basic statistics of single cell sequencing data are provided in Online-only Table 1. The cluster information and TCR typing data are presented in Supplementary File 1, which has also been uploaded to Figshare^27^.

**Table 2:** Statistics of somatic mutations detected by whole exome sequencing of CRC tumours.

Patient^a^	Frameshift insertion	Frameshift deletion	Frameshift substitution	Stopgain	Stoploss	Nonframeshift insertion	Nonframeshift deletion	Nonframeshift substitution	Missense SNV^b^	Synonymous SNV^b^	Unknown	Total
**P0123**	27	129	0	51	2	0	4	0	869	389	1	1,472
**P0825**	125	422	0	56	3	5	35	0	1,181	494	2	2,323
**P0909**	114	190	0	46	3	0	3	0	1,440	582	0	2,378
**P0413**	27	156	0	60	0	1	11	0	929	427	3	1,614
P0215	5	22	0	9	1	6	14	0	79	42	0	178
P0411	2	6	0	6	0	1	2	0	68	29	0	114
P0701	2	3	0	11	0	2	0	0	102	46	0	166
P1012	4	11	0	10	0	0	2	0	180	63	0	270
P1207	2	5	0	3	0	1	7	0	59	36	0	113
P1212	6	5	0	4	0	2	3	0	135	52	0	207
P1228	3	7	0	7	0	0	1	0	88	46	0	152
P0309	0	1	0	2	0	0	0	0	40	15	0	58

Somatic mutations were detected by variant caller Strelka and were annotated with ANNOVAR.

^a^MSI pateints are labelled in bold.

^b^SNV,single nucleotide variant.

## Technical Validation

### Validating the presence of tumour-infiltrating lymphocytes

**Fig. 5:**
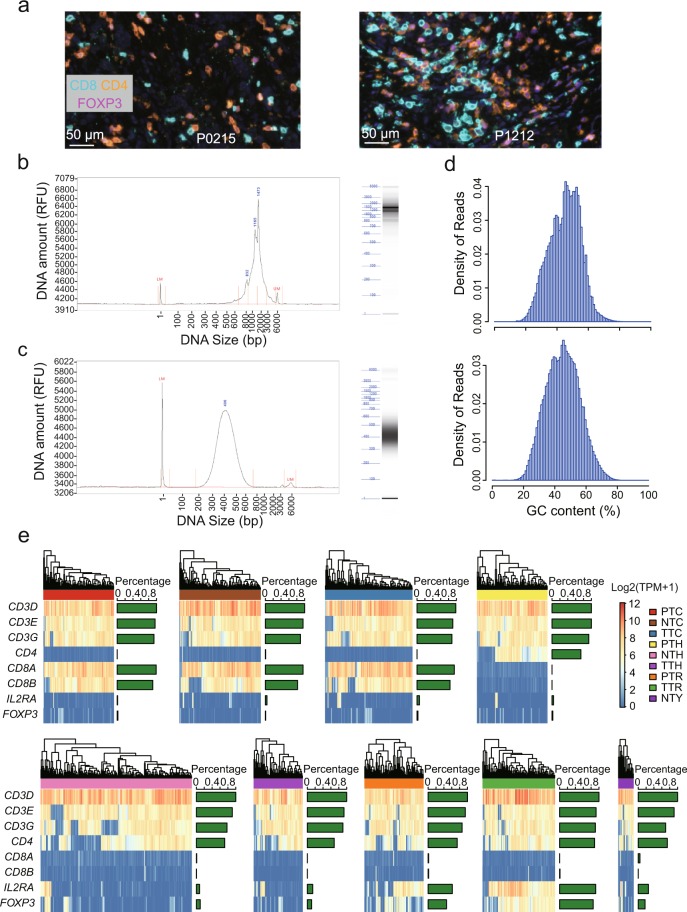
Quality assessment of single cell RNA-seq data. (**a**) Opal^TM^ multi-colour IHC staining to validate the existence of T cells in CRC tumours (exemplified by P0215 and P1212). (**b**) One representative example of cDNA size distribution derived from tumour of P0309. (**c**) One representative fragmentation profile of sequencing library after tagmentation prepared from pooled amplicons produced by PCR amplification of cDNA from samples of P0413. (**d**) The densities of GC content per sequence for two representative samples of P1212 and P1228. (**e**) Heatmaps demonstrating the expression levels of classic marker in each T cell subtypes. The right-sided barplots showed the percentages of cell with the expression of corresponding genes (TPM > 0). RFU, relative fluorescence unit.

Opal^TM^ multi-colour IHC staining were performed with anti-CD3, CD8,CD4, and FOXP3 antibodies to validate the existence of infiltrating T_C_, T_H_ and T_reg_ cells in tumour tissues (Fig. 5a).

### Validating the genomic features of CRC patients

Exome sequencing of bulk tumours from 12 patients showed that four patients harboured mutations in *TP53* and five patients harboured mutations in *APC*/*FBXW7*. These genomic alterations were consistent with the characteristics of colon adenocarcinoma (COAD) and rectum adenocarcinoma (READ) from The Cancer Genome Atlas (TCGA)^33^. Summarized tables were provided for the statistics of somatic mutations (Table 2) and selected cancer-associated somatic mutations (Online-only Table 2) that were detected in these patients.

### Validating the genomic alterations of MSI patients

Among the 12 CRC patients, 4 patients (P0123, P0909, P0825 and P0413) showed deficient in DNA mismatch repair based on IHC testing of four markers (MLH1, MSH2, MSH6, and PMS2)^11^, which was also supported by the much higher mutation load (Table 2). To further confirm the MSI status of these patients, we performed microsatellite instability testing by multiplex fluorescent PCR-based assay. Indeed, we found that 4 tumours from MSI patients were characterized by MSI-H phenotypes with two or more mononucleotide loci showing instability (Online-only Table 3).

### Validation of RNA samples & RNA-seq libraries

Quality control procedure was performed following the first round of purification of amplified cDNA products, including the detection of CD3D by qPCR and fragment analysis. For single cell samples with high quality (cycle threshold <30), the DNA products were further purified and the concentration of each sample was quantified (Fig. 5b). The constructed multiplex libraries were purified and pooled for quality assessment (Fig. 5c).

### Validating the quality of scRNA-seq data

Quality control analyses revealed that the raw sequence data were of high quality, with an average high-quality rate of 91.3% (Online-only Table 1). We assessed the qualities of clean data by statistics of per sequence quality scores and per sequence GC contents. For each sequence, an average of 87.9% bases have a quality score higher than phred quality 30 (Q30), and 94.5% bases have a quality score higher than phred quality 20 (Q20) (Online-only Table 1). In addition, the GC contents of each sample showed a similar normal distribution, with a mean value of 46.2% (Fig. 5d and Online-only Table 1). These statistics indicated that high-quality RNA-seq reads were obtained for downstream analysis.

### Validating cell types by marker genes

To evaluate the accuracy of FACS, we examined the expression of conventional marker genes of T cell subsets, including *CD3D*, *CD3E*, *CD3G*, *CD8A*, *CD8B*, *CD4*, *IL2RA* and *FOXP3* (Fig. 5e). While dropout event is prevalent and challenging in single cell RNA-seq data, the gene expression levels of classical T cell markers were consistent with protein levels measured by FACS. Specifically, all T cells were characterized by high expression of *CD3* genes (*CD3D*, *CD3E* and *CD3G*). Most T_C_ cells expressed high-level of *CD8* (*CD8A*, *CD8B*) but low-level of *CD4*, whereas T_H_ cells and T_regs_ exhibited the opposite pattern. T_regs_ showed high expressions of *IL2RA* encoding transmembrane protein CD25 and regulatory transcription factor *FOXP3* compared with T_H_ cells (Fig. 5e). Therefore, the expression patterns of classic T cell markers confirmed the reliability of T cell subtypes.

## Usage Notes

To facilitate reuse of our T cell dataset and broaden the user community, we developed a web server and will use the following sections to elaborate the design and functionalities provided by iSTARTRAC. iSTRATRAC is available at http://crctcell.cancer-pku.cn/.

### Design and implementation

Although we have provided an online portal at http://crc.cancer-pku.cn to depict gene expressions, only limited functionalities were presented, hindering the wide usage of our data. Here, to facilitate further exploration of our T cell data, we have developed a much enhanced web server iSTARTRAC to enable the comprehensive and customizable analyses.

The iSTARTRAC website is deployed on server with 64GB RAM and CPU Gold 6149 × 16 cores running the Ubuntu (version 16.04.4) Linux (version 4.4.0) operating system. The interface is constructed using the Shiny web application framework (version 1.2.0) in R (version 3.5.0) running on the Shiny-server (version 1.5.6.875).

iSTARTRAC is freely available to all users with no login requirement, and can be accessed by most web browsers including Google Chrome, Mozilla Firefox, Safari and Internet Explorer. The website automatically adjusts the look and feel according to different browsers and devices, but Google Chrome is recommended to achieve the best visualization.

### Sample options panel

In each module of iSTARTRAC, four categories of basic options are available for modulating the input samples of interest, including Cluster, Cell Type, Tissue Type and Patient. The Cluster icon consists of 20 clusters including 8 for CD8^+^ T cells and 12 for CD4^+^ T cells, and the Cell Type icon is composed of five cell types including CD8^+^ T cells, CD4^+^ T cells, CD4^+^ CD25^−^ T cells, CD4^+^ CD25^+^ T cells and CD4^+^ CD25^++^ T cells defined by FACS. Peripheral blood (P), adjacent normal (N) and tumour infiltrating (T) are included in the Tissue Type icon. The Patient icon contains eight MSS patients, as well as four MSI patients.

Moreover, iSTARTRAC presents interactive sliders that can be adjusted to change the dot sizes and line widths to achieve optimal visualization of the plots. Plots are regenerated on-the-fly as the user changes sliders or samples, providing an interactive experience that makes it possible to perform customizable analyses.

### Functionalities

iSTARTRAC provides key interactive and customizable functions including cluster visualization, gene expression demonstration, differential expression analyses between clusters or cell types, TCR sharing illustration, customizable analysis of STARTRAC indices and discrimination of differences between MSI and MSS patients (Fig. 4).

#### Cluster atlas

iSTARTRAC dynamically demonstrates the tSNE plot of cell clusters for user-defined T cells derived from given cell clusters, tissue origins, cell types and patients (in the ‘tSNE Plot’ tab). In addition, an annotation table of basic information of T cells is shown and users are allowed to download the table by clicking the DOWNLOAD button (in the ‘Table’ tab).

#### Gene expression

In this module, iSTARTRAC interactively plots expression distribution of a given gene in different clusters according to user-defined sample selections. The results can be presented in tSNE plot (in the ‘tSNE Plot’ tab), violin plots (in the ‘Violin Plot’ tab), or box plots (in the ‘Box Plot’ tab).

#### Differential expression analysis

iSTARTRAC performs differential expression (DE) analyses and identifies differentially expressed genes (DEGs) between any two given clusters (in ‘Cluster DEG’ tab) or cell types (in ‘Cell Type DEG’ tab), illustrating the results in volcano plots. Single cell transcriptome data is exceptionally appropriate for dissecting the intrinsic cellular heterogeneity. In addition to the commonly used unsupervised clustering, pairwise gene expression distribution, a simple and effective approach similar to FACS with proteins, can also be utilized to detect cell subpopulations. Accordingly, iSTARTRAC allows users to input a pair of genes to dynamically compartmentalize cell subpopulations and performs differential expression analysis for any two subdivided populations (in ‘*in silico* FACS’ tab). Users can adjust the thresholds of low/high-expression, as well as the significance thresholds of fold change and p-values after multiple testing adjustments. Furthermore, summary tables of signature gene for CD8^+^ and CD4^+^ T cells are provided and can be downloaded (in ‘Table’ tab).

#### TCR-based analysis

For any user-defined frequency of clonal cells, iSTARTRAC provides a tSNE plot to illustrate the distribution of clonal cells in each cluster, with non-clonal cells (cells harbouring TCRs with a frequency below the defined threshold) coloured in grey as background (in ‘tSNE Plot’ tab). The enormous TCR repertoire, which is essential for recognising foreign antigens and tumour neoantigens, could serve as tags to track T cell lineages. Accordingly, iSTARTRAC plots a heatmap to depict the TCR sharing patterns of various clusters enriched in different tissues (in ‘TCR Sharing’ tab), providing the clues of cross-tissue migration and state transition. In addition, iSTARTRAC presents bar plots to show the clonotype statistics of user-defined samples (in ‘Clonotype Statistics’ tab). A summary table of TCR typing is displayed and can be downloaded, which contains the information of TCR sequences and corresponding samples (in ‘Table’ tab).

#### STRATRAC indices

For given samples, iSTARTRAC dynamically illustrates the STRATRAC-dist indices to dissect the tissue preference of T cell clusters, yielding a discrete enrichment table decorated with colours (in ‘STARTRAC-dist’ tab). Users are allowed to adjust the thresholds for discretizing enrichment levels quantified by *R*_*o/e*_ (the ratio of observed over expected cell numbers in tissues to measure the enrichment of T cell clusters across different tissues). To reveal dynamic relationships of T cell subsets with respect to clonal expansion, migration and development transition, iSTARTRAC plots STRATRAC-expa/migr/tran indices for samples of user interest (in ‘STRATRAC-expa/migr/tran’ tab). Furthermore, pairwise STRATRAC-migr (in ‘pSTRATRAC-migr’ tab) and pairwise STRATRAC-tran (in ‘pSTRATRAC-tran’ tab) could also be dynamically illustrated according to user defined sample selections.

#### MSI versus MSS

With this module, users can delineate differences in term of cell compositions (in ‘Cell Percentage’ tab), STARTRAC indices (in ‘STARTRAC-expa/migr/tran’ tab) and gene expressions (in ‘DEG Analysis’ tab) between MSI and MSS patients for user-specified dataset of interest.

### Summary of scRNA-seq data application

The compendium dataset provided here, was produced primarily to illustrate the dynamic relationships of tumour-infiltrating lymphocytes in CRC, including functional states, clonal expansions, migrations and developmental transitions^11^.

The dataset can be further utilized to detect the transcript isoforms, non-coding transcripts and the potential splice variants. The differential isoform usages of T cell subtypes will shed new light on the underlying regulatory mechanisms of phenotypic differentiation and will provide opportunities for immuno-oncology modulation by determining the subtype specific expression of known and novel isoforms in TILs.

In addition, our dataset could serve as a resource for the comparison of different library preparation methods such as Smart-seq2 protocol and 10X platform, providing specific features of RNA-seq data produced with Smart-seq2 protocol.

The interactive platform, iSTARTRAC, could be explored by experimental biologists to dissect regulatory mechanisms of T cell differentiation, identify novel targets of immunotherapy, as well as to compare the differences of T cell compositions, gene expressions and STARTRAC indices between MSI and MSS patients. The comprehensive and customizable analyses with simple clicking through iSTARTRAC will facilitate data mining in cancer immunology community and help unleash the potential value of our CRC T cell data resource.

## Supplementary Information

### ISA-Tab metadata file

Download metadata file

### Supplementary Information

Supplementary File 1

## Data Availability

Sequencing data were processed using SAMtools (version 0.1.19), Picard (version 2.18.9) and GATK (version 3.8-1-0). Clean reads were aligned to human reference genome (hg19) using GSNAP (version 2014-10-22). TraCeR (version 2015-10-21) was used to assemble the TCR sequences of single T cells. All downstream analyses were performed using open source R (version 3.5.0). A series of R package were utilized for data analyses including HTSeqGenie (version 4.8.0) for expression quantification, single-cell consensus clustering (SC3, version 1.7.2) for unsupervised clustering and Rtsne (version 0.13) for dimension reduction. Static visualizations of iSTARTRAC are rendered as Portable Document Format (PDF). Tables are generated with R package DT (version 0.5), which provides R interface to the JavaScript library DataTables and allows for data querying, selection and download. Other R packages used by iSTARTRAC includes ggplot2 (version 3.1.0) for plotting box plots, violin plots and volcano plots, ComplexHeatmap (version 1.18.1) for plotting heatmaps, limma (version 3.36.5) for detecting DEGs, ks (version 1.11.3) for plotting cell densities, Startrac (version 0.1.0) for obtaining indices of STARTRAC, RColorBrewer (version 1.1-2) for colour palettes and org.Hs.eg.db (version 3.6.0) for converting gene names etc. Code for preliminary data processing including size-factor normalization, dimensional reduction and clustering is available on Figshare (10.6084/m9.figshare.8204624.v1), and code for STARTRAC is available on GitHub (https://github.com/Japrin/STARTRAC).

## Online-only Tables

**Online-only Table 1:** Sequencing data statistics of single T cells in CRC.

Patient	Cell type^a^	Average number of raw reads	Average number of raw bases	Average number of clean reads	Average number of clean bases	Average error rate of read1 (%)	Average error rate of read2 (%)	Average Q20 of read1 (%)	Average Q20 of read2 (%)	Average Q30 of read1 (%)	Average Q30 of read2 (%)	Average GC content of read1 (%)	Average GC content of read2 (%)	Average high quality rate (%)	Average uniquely mapped read pairs	Average mapping rate (%)	Average number of detected genes^b^	Number of cells
P0123	NP7	1,714,334	258,864,361	1,713,937	258,804,533	0.02	0.03	96.70	94.80	93.08	88.69	45.41	45.77	92.15	719,724	97.36	2,023	66
P0123	NTC	1,867,875	282,049,107	1,867,620	282,010,658	0.02	0.03	96.88	94.93	93.28	88.91	46.14	46.41	92.58	755,543	97.23	2,513	127
P0123	NTH	1,755,265	265,044,953	1,755,180	265,032,217	0.02	0.03	96.95	94.77	93.42	88.55	45.77	46.16	92.13	719,681	96.69	2,090	81
P0123	NTR	1,831,831	276,606,474	1,831,733	276,591,669	0.02	0.03	96.80	94.65	93.17	88.47	45.61	45.97	92.20	746,956	95.74	2,468	86
P0123	PP7	1,541,654	232,789,698	1,541,643	232,788,028	0.02	0.04	96.35	94.29	92.35	87.88	45.46	45.76	91.54	604,130	92.15	2,020	81
P0123	PTC	1,904,745	287,616,443	1,904,183	287,531,648	0.02	0.04	96.36	93.56	92.54	86.60	46.21	46.55	90.36	736,101	96.63	2,157	82
P0123	PTH	1,666,075	251,577,258	1,665,993	251,564,894	0.02	0.03	97.23	95.32	93.80	89.42	46.30	46.65	93.23	647,481	96.89	2,536	83
P0123	PTR	1,833,308	276,829,561	1,832,767	276,747,755	0.02	0.03	96.65	94.11	93.02	87.32	44.86	45.23	91.08	752,574	97.86	2,271	85
P0123	TP7	1,715,085	258,977,881	1,714,997	258,964,489	0.02	0.03	96.65	94.61	92.96	88.41	45.34	45.67	92.01	711,994	96.17	2,464	152
P0123	TTC	1,675,997	253,075,573	1,675,828	253,050,014	0.02	0.03	96.61	94.14	92.85	87.73	45.84	46.10	91.65	666,452	93.70	2,576	109
P0123	TTH	1,655,447	249,972,553	1,655,156	249,928,536	0.02	0.04	96.61	93.75	92.85	86.90	45.89	46.23	91.18	649,278	93.55	2,515	136
P0123	TTR	1,754,982	265,002,272	1,754,567	264,939,649	0.02	0.03	96.71	94.52	93.08	88.21	45.11	45.44	91.86	739,953	97.05	2,528	150
P0215	NTC	3,696,603	558,187,027	3,696,532	558,176,335	0.02	0.05	96.98	92.51	93.27	83.14	45.24	45.77	91.83	1,425,816	95.92	3,463	89
P0215	NTH	3,577,400	540,187,404	3,577,319	540,175,222	0.02	0.06	96.87	92.21	92.82	82.46	45.21	45.69	91.63	1,404,591	97.26	3,283	74
P0215	NTR	3,968,152	599,190,922	3,968,109	599,184,474	0.02	0.07	97.08	91.25	93.50	80.90	44.21	44.75	90.15	1,624,925	96.69	3,993	20
P0215	PTC	3,497,938	528,188,608	3,497,882	528,180,242	0.02	0.05	97.21	92.53	93.46	83.02	46.85	47.30	92.05	1,269,380	98.61	3,426	69
P0215	PTH	4,021,645	607,268,390	4,021,536	607,251,968	0.02	0.05	97.33	92.69	93.85	83.43	46.66	47.16	92.20	1,493,906	98.16	3,543	85
P0215	PTR	3,705,902	559,591,254	3,705,808	559,577,004	0.02	0.06	97.22	91.75	93.69	81.76	46.41	46.90	91.05	1,344,160	97.13	3,877	76
P0215	TTC	3,619,457	546,538,071	3,619,383	546,526,787	0.02	0.06	97.19	91.89	93.57	81.94	45.68	46.14	91.17	1,420,602	97.12	3,786	121
P0215	TTH	3,644,561	550,328,681	3,644,479	550,316,277	0.02	0.05	97.03	92.72	93.39	83.65	45.42	45.84	92.11	1,452,494	96.06	3,628	107
P0215	TTR	3,681,850	555,959,318	3,681,765	555,946,506	0.02	0.06	96.93	91.58	93.11	81.58	46.20	46.68	90.56	1,400,273	96.36	3,910	113
P0309	PP7	1,688,709	254,995,109	1,688,340	254,939,267	0.02	0.04	96.72	94.18	92.62	87.09	47.92	48.18	91.76	526,007	96.07	1,796	66
P0309	PTC	1,931,976	291,728,370	1,931,850	291,709,401	0.02	0.03	96.92	95.29	93.23	89.30	46.52	46.82	92.81	758,160	98.59	2,800	94
P0309	PTH	1,618,471	244,389,102	1,618,268	244,358,402	0.02	0.03	96.49	94.73	92.54	88.28	46.63	46.99	91.79	616,151	97.60	2,741	87
P0309	PTR	1,810,857	273,439,464	1,810,554	273,393,599	0.02	0.03	96.91	94.43	93.04	87.59	47.05	47.33	92.00	696,214	97.07	2,392	77
P0309	TP7	1,440,359	217,494,213	1,440,140	217,461,184	0.02	0.03	96.82	94.62	92.93	88.01	46.22	46.48	92.24	563,367	97.05	2,320	82
P0309	TTC	1,834,215	276,966,519	1,833,959	276,927,872	0.02	0.03	96.71	94.38	92.85	87.57	46.02	46.37	91.58	732,585	98.31	2,482	137
P0309	TTH	1,735,228	262,019,435	1,735,010	261,986,452	0.02	0.03	96.98	94.89	93.18	88.47	46.30	46.55	92.66	691,206	98.07	2,461	136
P0309	TTR	1,506,787	227,524,862	1,506,449	227,473,786	0.02	0.04	96.39	93.72	92.21	86.23	46.79	47.23	90.66	592,596	97.76	2,564	79
P0411	NTC	1,936,233	292,371,225	1,936,198	292,365,946	0.02	0.05	96.31	92.08	91.96	83.52	46.30	46.79	91.32	752,676	93.98	2,768	119
P0411	NTH	2,008,920	303,346,932	2,008,309	303,254,594	0.02	0.05	96.57	92.32	92.34	83.62	45.10	45.62	91.80	817,317	97.00	2,416	77
P0411	PTC	1,541,931	232,831,634	1,541,547	232,773,610	0.02	0.06	96.20	91.21	91.67	82.02	46.98	47.52	90.53	581,190	94.08	2,740	68
P0411	PTH	1,785,455	269,603,702	1,785,210	269,566,746	0.02	0.05	96.56	92.54	92.33	84.15	47.08	47.52	92.12	689,969	95.21	2,897	135
P0411	PTR	1,776,541	268,257,667	1,775,998	268,175,649	0.02	0.06	96.63	91.87	92.38	82.86	46.56	47.08	91.41	710,507	97.31	2,735	74
P0411	TTC	1,780,848	268,908,065	1,780,528	268,859,664	0.02	0.06	96.83	92.07	92.66	83.07	46.50	47.01	91.66	673,458	98.14	2,932	90
P0411	TTH	1,891,541	285,622,698	1,891,531	285,621,188	0.02	0.05	96.73	92.57	92.63	84.06	46.20	46.70	91.85	758,472	97.36	3,026	88
P0411	TTR	1,788,669	270,089,034	1,788,643	270,085,140	0.02	0.05	96.79	92.72	92.70	84.28	46.34	46.82	92.13	747,743	98.02	3,446	113
P0413	NTC	3,658,208	552,389,411	3,657,933	552,347,888	0.02	0.05	96.76	92.80	92.57	84.04	46.48	46.89	92.59	1,454,524	92.87	3,430	93
P0413	NTH	3,663,795	553,233,012	3,663,327	553,162,401	0.02	0.06	96.85	92.49	92.78	83.43	46.59	47.04	92.31	1,363,078	88.62	3,415	69
P0413	PTC	3,684,099	556,298,949	3,683,740	556,244,787	0.02	0.05	96.78	92.58	92.58	83.58	46.66	47.13	92.34	1,401,687	92.15	3,453	90
P0413	PTH	3,841,036	579,996,468	3,840,709	579,947,047	0.02	0.05	96.78	92.74	92.69	83.96	46.97	47.41	92.52	1,478,607	95.98	3,740	85
P0413	PTR	3,614,343	545,765,793	3,613,899	545,698,780	0.02	0.05	96.77	92.61	92.70	83.80	46.92	47.35	92.50	1,358,171	95.41	3,632	78
P0413	TTC	3,679,688	555,632,957	3,679,121	555,547,318	0.03	0.06	96.10	92.00	91.26	82.90	46.99	47.50	91.55	1,488,604	97.22	4,225	119
P0413	TTH	3,659,034	552,514,175	3,658,514	552,435,657	0.03	0.05	96.25	92.35	91.62	83.56	46.74	47.18	91.98	1,497,802	97.00	3,812	119
P0413	TTR	3,545,754	535,408,903	3,545,215	535,327,454	0.03	0.05	96.27	92.32	91.58	83.45	47.18	47.61	92.08	1,458,558	97.67	4,338	112
P0701	NTC	7,751,374	1,170,457,498	7,751,298	1,170,445,974	0.02	0.03	97.21	95.42	93.83	89.91	45.91	46.23	93.75	2,933,506	94.16	5,050	63
P0701	NTR	7,582,247	1,144,919,334	7,580,584	1,144,668,176	0.02	0.03	97.18	94.50	93.95	88.18	45.34	45.63	92.13	2,919,142	96.50	5,241	152
P0701	PTC	6,349,280	958,741,299	6,348,431	958,613,136	0.02	0.04	96.68	93.57	93.00	86.57	46.19	46.70	91.31	2,282,960	94.62	4,973	113
P0701	PTH	7,776,039	1,174,181,820	7,775,920	1,174,163,987	0.02	0.03	97.20	95.03	93.79	89.06	45.56	45.94	93.28	3,016,207	95.60	4,926	77
P0701	PTR	7,107,334	1,073,207,396	7,107,010	1,073,158,476	0.03	0.04	96.06	94.09	91.67	87.15	46.83	47.10	92.26	2,553,272	96.07	4,976	80
P0701	TTC	7,117,078	1,074,678,733	7,116,912	1,074,653,638	0.02	0.04	96.40	93.61	92.14	86.34	45.15	45.55	91.52	2,712,380	93.96	5,278	151
P0701	TTH	6,998,193	1,056,727,108	6,998,041	1,056,704,122	0.03	0.04	96.22	93.30	91.48	85.60	46.76	47.09	91.25	2,614,960	95.78	4,872	81
P0701	TTR	7,143,834	1,078,718,877	7,143,689	1,078,697,002	0.02	0.03	97.07	94.74	93.55	88.74	46.75	47.10	92.86	2,778,230	96.34	5,878	135
P0825	NTC	1,780,675	268,881,942	1,780,639	268,876,432	0.13	0.16	88.14	85.93	76.03	71.86	45.83	46.48	85.11	656,451	99.21	3,050	90
P0825	NTH	1,807,972	273,003,823	1,807,937	272,998,454	0.12	0.15	88.46	86.34	76.59	72.44	45.55	46.14	85.86	673,272	98.99	3,124	95
P0825	NTY	1,592,307	240,438,343	1,592,133	240,412,061	0.03	0.07	95.63	90.72	90.24	80.33	45.41	46.05	89.45	610,418	99.20	2,785	117
P0825	PTC	1,640,213	247,672,233	1,640,175	247,666,488	0.03	0.06	96.07	91.87	91.31	82.36	46.14	46.70	91.04	618,187	99.09	2,947	130
P0825	PTH	1,802,334	272,152,483	1,802,186	272,130,139	0.03	0.07	95.99	90.52	90.99	79.94	45.51	46.16	89.52	670,329	99.24	3,038	92
P0825	PTR	1,554,681	234,756,793	1,554,542	234,735,788	0.03	0.07	95.92	90.41	90.81	79.71	46.07	46.77	89.34	569,373	99.29	2,896	116
P0825	TTC	1,660,329	250,709,709	1,660,318	250,708,053	0.03	0.06	96.21	91.69	91.38	82.38	45.59	46.05	90.70	669,845	98.84	2,948	180
P0825	TTH	1,712,108	258,528,382	1,712,098	258,526,794	0.03	0.06	96.14	91.72	91.33	82.44	45.60	46.15	90.57	674,932	98.80	2,711	163
P0825	TTR	1,787,158	269,860,801	1,787,120	269,855,136	0.03	0.06	96.16	91.64	91.41	82.03	46.11	46.64	90.82	709,066	98.80	3,374	174
P0825	TTY	1,625,054	245,383,201	1,624,843	245,351,299	0.03	0.07	95.57	90.84	90.19	80.46	45.44	46.19	89.60	658,468	99.37	2,663	96
P0909	NTC	3,110,820	469,733,749	3,110,564	469,695,119	0.02	0.03	97.04	94.43	93.62	87.93	45.43	45.52	91.75	1,179,709	95.32	3,668	47
P0909	NTH	3,736,288	564,179,461	3,735,299	564,030,116	0.02	0.03	97.23	94.83	93.88	88.78	45.84	45.92	92.69	1,426,221	96.12	3,918	148
P0909	PTC	3,504,476	529,175,889	3,504,352	529,157,097	0.02	0.04	97.05	94.26	93.43	87.48	45.98	46.02	92.28	1,341,780	93.40	4,026	72
P0909	PTH	3,981,055	601,139,293	3,980,673	601,081,621	0.02	0.04	97.13	94.22	93.64	87.45	45.75	45.82	92.03	1,512,970	95.04	3,953	85
P0909	PTR	3,329,581	502,766,675	3,329,434	502,744,516	0.02	0.04	96.52	92.40	92.62	84.40	45.32	45.52	89.56	1,239,372	92.42	3,961	67
P0909	PTY	1,716,265	259,155,966	1,716,067	259,126,052	0.03	0.07	96.29	90.60	91.53	79.95	45.60	46.26	90.30	648,357	94.99	3,086	77
P0909	TTC	3,869,919	584,357,827	3,869,131	584,238,841	0.02	0.03	97.61	95.76	94.76	90.60	45.56	45.76	94.01	1,618,091	97.50	4,694	139
P0909	TTH	3,815,422	576,128,734	3,814,945	576,056,626	0.02	0.03	97.21	94.87	93.88	88.86	45.54	45.60	92.99	1,589,333	97.23	4,596	214
P0909	TTR	3,666,375	553,622,636	3,665,775	553,532,004	0.02	0.03	97.32	94.94	94.06	88.85	45.71	45.78	92.70	1,537,775	96.91	4,319	171
P0909	TTY	1,702,643	257,099,045	1,702,439	257,068,344	0.02	0.06	96.50	92.03	91.88	82.30	45.48	46.01	91.83	698,722	97.23	3,429	85
P1012	PTC	3,748,214	565,980,295	3,748,164	565,972,815	0.02	0.06	96.75	91.78	92.60	82.04	46.04	46.27	90.75	1,491,544	97.56	4,053	95
P1012	PTH	3,838,306	579,584,155	3,838,199	579,568,083	0.02	0.05	96.94	92.55	92.83	83.22	46.08	46.28	91.94	1,525,269	97.92	3,995	88
P1012	PTR	3,162,102	477,477,470	3,162,008	477,463,136	0.02	0.08	96.74	90.22	92.38	79.04	45.91	46.18	88.99	1,220,352	98.33	3,715	84
P1012	PTY	1,756,369	265,211,703	1,756,163	265,180,660	0.03	0.07	96.36	91.05	91.67	80.68	45.78	46.44	90.74	680,840	95.97	3,272	87
P1012	TTC	3,702,952	559,145,801	3,702,437	559,067,940	0.03	0.07	96.62	91.25	92.13	80.83	46.37	46.61	90.13	1,489,473	97.76	4,193	241
P1012	TTH	3,411,715	515,168,953	3,411,494	515,135,622	0.02	0.07	96.45	90.79	91.83	80.16	46.13	46.39	89.59	1,304,416	96.28	3,430	170
P1012	TTR	3,425,917	517,313,415	3,425,794	517,294,969	0.02	0.06	96.96	91.57	92.81	81.48	46.60	46.81	90.78	1,362,675	97.56	4,274	177
P1012	TTY	1,905,667	287,755,716	1,905,445	287,722,243	0.03	0.07	96.36	90.83	91.72	80.30	45.82	46.49	90.33	757,246	96.45	3,122	123
P1207	PTC	3,606,923	544,645,331	3,605,187	544,383,216	0.02	0.06	96.55	91.51	92.51	81.90	45.66	46.18	90.24	1,389,714	93.68	3,747	126
P1207	TTC	3,540,183	534,567,647	3,538,500	534,313,532	0.03	0.09	95.66	88.48	90.99	77.49	46.83	47.41	86.62	1,241,982	87.04	3,774	84
P1212	NTC	3,767,875	568,949,118	3,766,095	568,680,405	0.03	0.07	96.15	90.44	91.36	79.52	45.61	46.12	89.70	1,443,907	93.03	3,967	205
P1212	NTH	3,561,632	537,806,501	3,560,120	537,578,055	0.03	0.08	96.22	90.13	91.17	78.33	46.15	46.69	89.83	1,427,653	97.65	3,708	225
P1212	NTY	4,062,957	613,506,540	4,060,649	613,157,953	0.03	0.07	96.64	91.43	92.10	80.68	46.31	46.72	91.19	1,650,570	97.46	4,199	23
P1212	PTC	3,801,176	573,977,643	3,799,275	573,690,503	0.03	0.09	96.05	89.18	90.92	77.03	46.01	46.54	88.56	1,410,374	93.67	4,345	105
P1212	PTH	3,724,347	562,376,414	3,722,518	562,100,151	0.03	0.08	96.36	90.62	91.39	79.15	46.25	46.77	90.39	1,474,677	97.65	4,083	105
P1212	PTR	3,810,787	575,428,857	3,809,226	575,193,198	0.03	0.08	96.41	90.89	91.52	79.62	46.45	46.95	90.65	1,511,475	95.94	4,137	89
P1212	TTC	3,690,325	557,239,091	3,688,586	556,976,512	0.03	0.08	96.28	89.99	91.55	78.68	45.91	46.42	89.15	1,419,152	92.66	4,119	211
P1212	TTH	3,549,079	535,910,890	3,547,181	535,624,366	0.03	0.09	96.25	89.41	91.03	76.87	46.28	46.80	88.93	1,425,366	98.98	3,909	73
P1212	TTR	3,700,772	558,816,511	3,698,983	558,546,384	0.03	0.10	95.88	88.84	90.72	76.64	45.88	46.47	87.96	1,404,682	93.37	4,800	128
P1228	NTC	3,805,123	574,573,567	3,804,741	574,515,827	0.02	0.05	96.90	93.42	92.96	85.24	45.92	46.39	93.34	1,538,977	97.84	3,933	239
P1228	NTH	3,914,655	591,112,903	3,914,494	591,088,561	0.03	0.07	96.62	92.17	92.33	82.80	45.66	46.17	93.42	1,642,232	98.11	3,589	184
P1228	NTR	3,825,729	577,685,132	3,825,711	577,682,320	0.02	0.05	96.66	93.11	92.48	84.73	45.41	45.91	93.02	1,594,162	96.81	3,729	148
P1228	PTC	3,559,129	537,428,541	3,558,953	537,401,927	0.03	0.10	95.77	89.66	90.64	78.61	45.77	46.45	91.59	1,424,504	96.10	4,061	88
P1228	PTH	3,771,713	569,528,705	3,771,635	569,516,855	0.02	0.05	97.06	93.37	93.38	85.03	45.40	45.92	93.42	1,550,108	98.34	4,209	75
P1228	PTR	3,632,355	548,485,679	3,632,307	548,478,403	0.02	0.05	96.86	93.48	92.89	85.28	45.57	46.05	93.45	1,515,595	98.20	4,142	86
P1228	TTC	3,862,801	583,282,997	3,862,477	583,234,071	0.03	0.06	96.49	91.64	91.97	81.98	45.78	46.35	91.00	1,608,945	98.58	3,449	224
P1228	TTH	4,020,957	607,164,458	4,020,253	607,058,128	0.02	0.04	96.85	94.01	93.00	86.18	44.48	45.05	93.93	1,714,680	98.55	3,484	83
P1228	TTR	3,503,527	529,032,593	3,503,226	528,987,104	0.03	0.06	96.12	91.46	91.39	81.78	44.75	45.37	90.91	1,437,129	96.99	4,234	83

^a^PTC, CD8^+^ cytotoxic T cells from peripheral blood; TTC, CD8^+^ cytotoxic T cells from tumour tissue; NTC, CD8^+^ cytotoxic T cells from adjacent normal tissue.

PTH, CD4^+^CD25^−^ cells from peripheral blood; TTH, CD4^+^CD25^−^ cells from tumour tissue; NTH, CD4^+^CD25^−^ cells from adjacent normal tissue.

PTR, CD4^+^CD25^hi^ cells from peripheral blood; TTR, CD4^+^CD25^hi^ cells from tumour tissue; NTR, CD4^+^CD25^hi^ cells from adjacent normal tissue.

PTY, CD4^+^CD25^int^ cells from peripheral blood; TTY, CD4^+^CD25^int^ cells from tumour tissue; NTY, CD4^+^CD25^int^ cells from adjacent normal tissue.

PP7, CD4^+^ T cells from peripheral blood; TP7, CD4^+^ T cells from tumour tissue; NP7, CD4^+^ T cells from adjacent normal tissue.

^b^A gene was defined as “detected” if the number of mapped read pairs of this gene was larger than 0.

**Online-only Table 2:** Selected cancer-associated somatic mutations detected in CRC tumours.

Patient^a^	Genomic mutation	Exonic function^b^	Gene	c_DNA mutation	Protein mutation	Hot spot	Driver gene^c^
P1207	12:25398284,C>T	missense_SNV	KRAS	c.G35A	p.G12D	Yes	Oncogene
	17:56448303,G>GC	frameshift_insertion	RNF43	c.343dupG	p.A115fs	No	TSG
	17:56492719,C>A	stopgain	RNF43	c.G220T	p.E74X	No	TSG
	18:48591870,TGCCCTATTG>T	nonframeshift_deletion	SMAD4	c.569_577del	p.190_193del	No	TSG
	19:11132513,C>T	missense_SNV	SMARCA4	c.C338T	p.T113M	Yes	TSG
	20:57429320,G>A	missense_SNV	GNAS	c.G1000A	p.G334S	Yes	Oncogene
P1212	17:7578440,T>C	missense_SNV	TP53	c.A13G	p.K5E	Yes	TSG
P1228	3:41278180,G>A	missense_SNV	CTNNB1	c.G2056A	p.E686K	No	Oncogene
	5:112175617,TC>T	frameshift_deletion	APC	c.2227delC	p.P743fs	Yes	TSG
	22:24159001,G>T	missense_SNV	SMARCB1	c.G673T	p.D225Y	No	TSG
P0215	4:153332832,G>A	stopgain	FBXW7	c.C124T	p.Q42X	No	TSG
	5:112174631,C>T	stopgain	APC	c.C1240T	p.R414X	Yes	TSG
	5:112175174,G>T	stopgain	APC	c.G1783T	p.E595X	Yes	TSG
P0411	17:7577046,C>A	stopgain	TP53	c.G415T	p.E139X	Yes	TSG
	17:70119882,A>AC	frameshift_insertion	SOX9	c.885dupC	p.D295fs	No	TSG
**P0413**	1:43804331,G>T	missense_SNV	MPL	c.G331T	p.V111L	No	Oncogene
	3:41275757,C>T	missense_SNV	CTNNB1	c.C1652T	p.T551M	No	Oncogene
	3:47158201,C>T	missense_SNV	SETD2	c.G4498A	p.E1500K	No	TSG
	3:128205864,G>A	missense_SNV	GATA2	c.C11T	p.A4V	No	Oncogene
	3:138665368,G>A	missense_SNV	FOXL2	c.C197T	p.A66V	No	Oncogene
	3:178952088,A>G	missense_SNV	PIK3CA	c.A3143G	p.H1048R	Yes	Oncogene
	9:110249887,A>G	missense_SNV	KLF4	c.T638C	p.V213A	No	Oncogene
	11:108114816,CT>C	frameshift_deletion	ATM	c.634delT	p.F212fs	Yes	TSG
	12:46123836,TA>T	frameshift_deletion	ARID2	c.103delA	p.K35fs	No	TSG
	14:81422170,G>A	missense_SNV	TSHR	c.G146A	p.S49N	No	Oncogene
	16:348044,C>T	missense_SNV	AXIN1	c.G1462A	p.G488R	No	TSG
	16:3801727,G>A	missense_SNV	CREBBP	c.C3665T	p.T1222M	No	TSG
	17:7577538,C>T	missense_SNV	TP53	c.G266A	p.R89Q	Yes	TSG
	17:56435160,AC>A	frameshift_deletion	RNF43	c.1853delG	p.G618fs	Yes	TSG
	18:42531605,C>T	missense_SNV	SETBP1	c.C2300T	p.S767L	No	Oncogene
	19:17942557,G>A	missense_SNV	JAK3	c.C2731T	p.R911C	No	Oncogene
**P0825**	1:27100983,C>T	missense_SNV	ARID1A	c.C803T	p.S268F	No	TSG
	1:27105659,C>T	missense_SNV	ARID1A	c.C254T	p.A85V	No	TSG
	1:27105930,TG>T	frameshift_deletion	ARID1A	c.268delG	p.G90fs	Yes	TSG
	2:48026881,G>A	missense_SNV	MSH6	c.G853A	p.A285T	No	TSG
	3:178947836,A>G	missense_SNV	PIK3CA	c.A2711G	p.Y904C	No	Oncogene
	4:55594093,C>T	missense_SNV	KIT	c.C226T	p.P76S	No	Oncogene
	4:106155778,G>GA	frameshift_insertion	TET2	c.680dupA	p.E227fs	No	TSG
	5:56177480,G>T	missense_SNV	MAP3K1	c.G2453T	p.R818M	No	TSG
	5:112154771,C>T	stopgain	APC	c.C988T	p.R330X	Yes	TSG
	7:2968322,CG>C	frameshift_deletion	CARD11	c.1663delC	p.R555fs	Yes	Oncogene
	7:140453136,A>T	missense_SNV	BRAF	c.T1799A	p.V600E	Yes	Oncogene
	9:98270529,GC>G	frameshift_deletion	PTCH1	c.114delG	p.G38fs	No	TSG
	16:50813641,C>T	missense_SNV	CYLD	c.C1195T	p.L399F	No	TSG
	17:29667635,T>C	missense_SNV	NF1	c.T1598C	p.L533S	No	TSG
	17:29676257,G>A	missense_SNV	NF1	c.G1873A	p.A625T	No	TSG
	17:56435160,AC>A	frameshift_deletion	RNF43	c.1853delG	p.G618fs	Yes	TSG
	19:42796882,G>GC	frameshift_insertion	CIC	c.3341dupC	p.A1114fs	Yes	TSG
	20:4167411,C>T	stopgain	SMOX	c.C1552T	p.Q518X	No	Oncogene
	20:57415505,C>T	missense_SNV	GNAS	c.C344T	p.T115I	No	Oncogene
	21:44513265,G>A	missense_SNV	U2AF1	c.C451T	p.R151W	No	Oncogene
**P0123**	1:65325832,CG>C	frameshift_deletion	JAK1	c.1289delC	p.P430fs	Yes	Oncogene
	1:120548005,C>A	missense_SNV	NOTCH2	c.G113T	p.C38F	No	TSG
	3:37035079,C>T	missense_SNV	MLH1	c.C41T	p.T14I	No	TSG
	3:178952085,A>G	missense_SNV	PIK3CA	c.A3140G	p.H1047R	Yes	Oncogene
	4:153247303,T>C	missense_SNV	FBXW7	c.A971G	p.H324R	Yes	TSG
	5:112174898,G>T	stopgain	APC	c.G1507T	p.G503X	No	TSG
	6:33286928,G>T	missense_SNV	DAXX	c.C1784A	p.P595H	No	TSG
	6:157505442,GA>G	frameshift_deletion	ARID1B	c.3385delA	p.K1129fs	No	TSG
	7:140453136,A>T	missense_SNV	BRAF	c.T1799A	p.V600E	Yes	Oncogene
	9:21974705,G>A	missense_SNV	CDKN2A	c.C122T	p.P41L	No	TSG
	13:32954022,CA>C	frameshift_deletion	BRCA2	c.9090delA	p.T3030fs	Yes	TSG
	19:1221306,G>A	missense_SNV	STK11	c.G829A	p.D277N	No	TSG
	19:42793222,G>A	missense_SNV	CIC	c.G1114A	p.A372T	No	TSG
	20:31024242,C>T	stopgain	ASXL1	c.C3400T	p.Q1134X	No	TSG
	X:76938647,G>A	missense_SNV	ATRX	c.C1987T	p.R663C	Yes	TSG
P0701	4:153244185,G>A	stopgain	FBXW7	c.C1444T	p.R482X	Yes	TSG
	4:153247289,G>A	missense_SNV	FBXW7	c.C985T	p.R329C	Yes	TSG
	5:112174094,T>TA	stopgain	APC	c.704dupA	p.Y235_N236delinsX	Yes	TSG
	5:112175507,C>T	stopgain	APC	c.C2116T	p.Q706X	Yes	TSG
	11:108202177,G>A	missense_SNV	ATM	c.G2593A	p.G865R	No	TSG
	17:70119805,CT>C	frameshift_deletion	SOX9	c.808delT	p.F270fs	No	TSG
**P0909**	1:27106105,C>T	missense_SNV	ARID1A	c.C442T	p.R148W	No	TSG
	2:29416773,T>C	missense_SNV	ALK	c.A976G	p.N326D	No	Oncogene
	3:52610644,T>C	missense_SNV	PBRM1	c.A3508G	p.T1170A	No	TSG
	4:153244155,TC>T	frameshift_deletion	FBXW7	c.1473delG	p.G491fs	Yes	TSG
	5:112173917,C>T	stopgain	APC	c.C526T	p.R176X	Yes	TSG
	9:139395150,T>C	missense_SNV	NOTCH1	c.A5788G	p.T1930A	No	TSG
	10:123276800,GA>G	frameshift_deletion	FGFR2	c.828delT	p.F276fs	No	Oncogene
	11:108186742,C>T	stopgain	ATM	c.C1171T	p.R391X	Yes	TSG
	11:119077219,A>T	missense_SNV	CBL	c.A92T	p.D31V	No	Oncogene
	12:46245445,C>T	missense_SNV	ARID2	c.C1541T	p.T514M	No	TSG
	13:28589318,T>C	missense_SNV	FLT3	c.A2606G	p.Q869R	No	Oncogene
	15:45007681,T>C	missense_SNV	B2M	c.T128C	p.L43P	Yes	TSG
	15:45007824,A>AC	frameshift_insertion	B2M	c.272dupC	p.T91fs	No	TSG
	15:90631688,A>AT	frameshift_insertion	IDH2	c.190dupA	p.M64fs	No	Oncogene
	16:3781375,G>A	missense_SNV	CREBBP	c.C4876T	p.R1626C	Yes	TSG
	19:11144117,T>C	missense_SNV	SMARCA4	c.T1307C	p.M436T	No	TSG
	19:42795608,AC>A	frameshift_deletion	CIC	c.2689delC	p.P897fs	No	TSG
	22:41556705,A>G	missense_SNV	EP300	c.A3650G	p.D1217G	No	TSG
	22:41574697,T>TC	frameshift_insertion	EP300	c.6983dupC	p.S2328fs	No	TSG
	X:63411935,T>TC	frameshift_insertion	AMER1	c.1231dupG	p.E411fs	No	TSG
P1012	7:140453136,A>T	missense_SNV	BRAF	c.T1799A	p.V600E	Yes	Oncogene
	9:139402561,C>G	missense_SNV	NOTCH1	c.G1046C	p.G349A	No	TSG
	17:7577082,C>T	missense_SNV	TP53	c.G379A	p.E127K	Yes	TSG
	18:48581243,C>T	stopgain	SMAD4	c.C82T	p.Q28X	Yes	TSG

^a^MSI pateints are labelled in bold.

^b^SNV, single nucleotide variant.

^c^TSG, tumour suppressor gene.

**Online-only Table 3:** DNA fragment sizes of short tandem repeat loci from tested patients in microsatellite instability testing experiment.

		Normal DNA			Tumour DNA			
Patient^a^	Marker^b^	Size 1	Size 2	Size 3	Size 1	Size 2	Size 3	Size 4
**P0909**	NR21	106.09			106.02	96.76		
	Bat26	177.31			166.03	161.95	177.4	
	Bat25	118.5			118.47	111.84		
	NR24	134.3			134.2	127.87		
	Mono27	170.33	172.23		170.42	160.49	172.2	
	PentaC	230.4			230.4	204.36		
	PentaD	172.39	176.99		177.08	172.37	181.97	
**P0413**	NR21	107.09			103.35	98.66	107.16	
	Bat26	178.46			172.26	169.19	178.38	
	Bat25	119.43			119.44	114.73		
	NR24	134.2			134.2	127.9		
	Mono27	172.23			172.17	165.05		
	PentaC	230.5	251.38		230.46	225.27	240.89	251.35
	PentaD	181.93	191.65		191.65	181.93		
**P0825**	NR21	105.55			105.57	103.69	98.12	
	Bat26	178			178	166.76	163.62	
	Bat25	119.71			119.8	114.19	111.42	
	NR24	134.06			134.06	128.7	126.06	
	Mono27	172.95			173.05	164.95	163.24	
	PentaC	230.28			230.29			
	PentaD	176.57	186.2		176.67	186.33		
**P0123**	NR21	106.67			101.93	100.1	106.66	
	Bat26	178.16			173.98	167.87	178.15	
	Bat25	118.96			119.04	112.53		
	NR24	134.03			134.08	130.5		
	Mono27	173.09			173.06	167.69		
	PentaC	230.26				230.26		
	PentaD	176.77	196.07		196.03	176.76		
P1212	NR21	106.11			106.2			
	Bat26	178.46			178.37			
	Bat25	119.51			119.49			
	NR24	133.21			133.37			
	Mono27	169.55			169.43			
	PentaC	225.37	230.56		225.3	230.42		
	PentaD	176.93	206.02		177.07	206.15		
P0215	NR21	105.97			105.93			
	Bat26	178.11			178.12			
	Bat25	119.28			119.11			
	NR24	133.66			133.47			
	Mono27	173.29			173.17			
	PentaC	219.8	230.25		219.8	230.17		
	PentaD	176.39	190.96		176.25	190.78		
P0701	NR21	106.99			107.16			
	Bat26	177.31			177.26			
	Bat25	119.5			119.33			
	NR24	133.32			133.36			
	Mono27	172.23			172.27			
	PentaC	220.01	230.42		219.99	230.41		
	PentaD	191.54			191.64			
P1012	NR21	106.01			106.09			
	Bat26	177.4			177.44			
	Bat25	119.32			119.33			
	NR24	133.31			133.32			
	Mono27	172.2			172.17			
	PentaC	230.52	235.67		230.44	235.62		
	PentaD	191.64			191.62			
P1207	NR21	105.96			106.09			
	Bat26	178.4			178.4			
	Bat25	119.33			119.33			
	NR24	133.43			133.26			
	Mono27	170.41	172.33		170.4	172.16		
	PentaC	230.53	235.74		230.42	235.61		
	PentaD	172.49	191.71		172.32	191.71		

MSI pateints are labelled in bold.

PentaC and PantaD, two much less variable pentanucleotide repeats.
